# Supplementary material for: Detection of O25B-ST131 clone and blaCTX-M-15 gene in Escherichia coli isolated from patients with COVID-19
Source: PeerJ. 2025 Mar 6;13:e19011. doi: 10.7717/peerj.19011 (PMC11890292; doi:10.7717/peerj.19011)
Supplement: Supplemental Information 1 [file peerj-13-19011-s001.docx]

**Supplementary Data 1A.** Incidence of Bacterial isolated from various collected samples from infected patients.

| **Type of sample** | ***E.coli*** | **Klebsiella** | **Other Gram negative** | **Pseudomonas** | **Non fermenters** | ***Staphylococcus aureus*** | **Other Gram positive** | **Fungus** | **No growth** |
| --- | --- | --- | --- | --- | --- | --- | --- | --- | --- |
| **Sputum** | 44 | 34 | 11 | - | 2 | - | 15 | 2 | 41 |
| **ETA** | 32 | 12 | 2 | 1 | 7 | 5 | 8 | 4 | 14 |
| **BAL** | 11 | 5 | 6 | - | 1 | 1 | 2 | 3 | 12 |
| **Blood** | 11 | 2 | 2 | - | - | 17 | - | 1 | 20 |
| **Urine** | 126 | 4 | 5 | 13 | - | - | - | 3 | 11 |
| **Catheter tip + CV line** | 3 | - | 2 | - | - | 9 | - | - | 3 |
| **Nasal** | 1 | - | 3 | - | - | 4 | - | 2 | 2 |
| **Fluid aspirate** | 6 | - |  | - | - | 3 | - | - | - |
| **Total** | 234 | 57 | 31 | 14 | 10 | 39 | 25 | 15 | 103 |
